# Supplementary material for: Are carbon emissions trading and green financial instruments synergistic? -Comprehensive quantitative research based on content analysis
Source: PLoS One. 2024 Mar 7;19(3):e0298601. doi: 10.1371/journal.pone.0298601 (PMC10919601; doi:10.1371/journal.pone.0298601)
Supplement: S1 Data — (ZIP) [file pone.0298601.s001.zip › Data/地级市Z_F工作报告.zip!/╕╩╦α/░╫╥°╩╨/└┤╫╘╣1⁄2╓┌║┼í╛┬φ┐╦╩2╛▌═°í┐_2016.docx]

各位代表：

现在，我代表市人民政府向大会作工作报告，请予审议，并请政协委员和列席人员提出意见。

“十二五”及2015年工作回顾

“十二五”时期，面对复杂多变的宏观经济环境和艰巨繁重的转型发展任务，全市上下认真贯彻落实中央和省委、省政府及市委的一系列决策部署，紧紧围绕转变发展方式这条主线，主动适应经济发展新常态，扎实推进稳增长、调结构、促改革、惠民生各项工作，基本实现经济社会发展各项目标，为全面建成小康社会奠定了坚实基础。

过去五年，是经济运行稳中有进、综合实力不断增强的五年。我们始终把稳增长作为主攻方向，及时分析困难，研究对策，攻坚克难，有力保障了经济平稳增长。全市生产总值年均增长11.2%，人均生产总值年均增长12%，工业增加值年均增长13.1%，固定资产投资年均增长20.2%，社会消费品零售总额年均增长15.6%，一般公共预算收入年均增长16.6%，城乡居民人均可支配收入年均分别增长11.8%、13.8%。

过去五年，是产业结构调整优化、转型升级取得突破的五年。我们始终保持发展定力，把转方式、调结构作为转型发展的根本举措,着力培育新的增长动能，经济增长质量和效益逐步提高。三次产业比例由12.1:55:32.9调整到13.6:44.7:41.7。粮食产量实现“十二连增”,“三品一标”农产品认证面积达到234.4万亩，农业产业化龙头企业由190家发展到365家，农民专业合作社由618家发展到7147家，农产品加工转化率由42%提高到52%。“3+4”产业集群发展，完成工业投资201.7亿元，是“十一五”时期的2倍，战略性新兴产业增加值占生产总值比重由6%提高到10%。循环经济发展成效显著，全市工业固体废物综合利用率达到75%，淘汰落后产能395.2万吨，资源产出率、能源产出率每吨分别提高756元、1710元。文化旅游融合发展，文化企业由410家增加到832家，文化产业增加值年均增长30.5%，旅游综合收入年均增长25%。

过去五年，是发展基础不断夯实、城乡面貌显著变化的五年。我们始终把打基础、谋长远作为可持续发展的基本要求，着力加大基础设施建设力度，发展环境得到明显改善。新增农村公路（硬化）3196公里，建制村公路通畅率达到83%。营双高速公路景泰段建成通车。新增高标准基本农田56.4万亩。建成双永供水和121处农村饮水安全工程，解决了68.9万人饮水不安全问题。中心城区新改建道路56.4公里，建成区由84.6平方公里扩大到99平方公里，生活污水处理率达到83.3%，生活垃圾无害化处理率达到92.7%，人均绿地达到8.1平方米。实施矿山环境治理、工业污染源综合治理、重金属污染治理、东大沟流域河道综合整治等工程，生态环境不断改善。市区空气质量持续好转，2015年优良天数按新标准达到280天。白银工业集中区“一区六园”完成基础设施投资57.3亿元，园区平台支撑作用逐步增强。

过去五年，是改革创新逐步深化、发展活力持续释放的五年。我们始终把改革创新作为经济社会发展的强大动力源，努力破除束缚发展的体制机制障碍，经济发展活力有效激发。以简政放权释放红利,完成“四单一网”建设，取消、下放、调整审批事项488项，全部取消非行政事业性收费，清理规范行政审批中介机构142家；组建公共资源交易中心，工程建设、政府采购、国有产权交易、土地矿产出售等全部纳入集中统一监管。通过市场化改革催生经济活力，市场主体增长1.2倍,注册资本增长4倍。围绕重点领域推进改革，靖煤集团整体重组刘化集团，煤矿企业由57家整合至32家，白银公司供热供水系统和白银公司、银光公司职工医院完成移交；233家企业在区域性股权交易中心挂牌，“三权”抵押贷款余额3.6亿元；在全省率先开展农业设施确权工作，流转土地113.5万亩。以园区为平台推动自主创新，兰白科技创新改革试验区、白银国家农业科技园区获科技部批准，科技企业孵化器被认定为国家级孵化器，白银公司被认定为国家技术创新示范企业，世界首座超导变电站在高新区建成。全市万人发明专利拥有量达到1.28件，科技对经济贡献率由45%提高到51%，我市成为全国科技进步先进市、全国知识产权试点城市。

过去五年，是社会事业持续发展、民生保障不断提高的五年。我们始终坚持民生优先政策取向，投入民生资金471.9亿元，占一般公共预算支出的84.2%。城乡收入比由3.6:1缩小到3.3:1。推进“1236”扶贫攻坚和“双联”行动，农村贫困人口由54.78万人下降到23.6万人，贫困发生率由41%下降到17.7%。优先发展教育事业，新改扩建校舍129.6万平方米，乡镇中心幼儿园实现全覆盖并向行政村延伸，义务教育通过国家“两基”验收，巩固率达到99.4%，基本普及高中阶段教育，公民平均受教育年限达到10.5年。成立白银矿冶职业技术学院，填补了我市普通高校的空白。深化医药卫生体制改革，省中医院托管共建市中西医结合医院，市残疾人康复中心挂牌成立，贫困村标准化卫生室实现全覆盖。实施广播电视“村村通”、“户户通”工程，覆盖农户22.8万户。建成“乡村舞台”395个,“农家书屋”实现行政村全覆盖。成功举办全国男子自由式摔跤锦标赛、全省第十三届运动会、第九届残运会等大型体育赛事，新建“一村一场”430个、乡镇及社区体育健身中心30个，人均体育场地由0.9平方米增加到1.2平方米。完善城乡社保体系,城市和农村低保人均月补助分别提高213元、70元,农村五保供养标准提高2514元,新农合、城市居民医保人均筹资标准分别提高320元、220元，建成农村互助老人幸福院、社区日间照料中心404所。改善居民居住条件,建成保障性安居工程4.35万套，改造农村危旧房6.8万户。推进“平安白银”建设，加强安全生产监管，维护了公共安全和社会稳定。

各位代表！刚刚过去的2015年，是“十二五”收官之年。在经济下行压力加大的情况下，我们科学把握发展趋势，深入实施各项改革措施，加快调整经济结构，突出保障和改善民生，较好地完成了市八届人大四次会议确定的各项目标任务。全市完成生产总值434.27亿元，同比增长6.8%；规上工业增加值136.95亿元，增长4.8%；固定资产投资474.46亿元，增长10.74%；社会消费品零售总额177.65亿元，增长8.7%；居民消费价格总水平上涨0.3%；城镇居民人均可支配收入23438元，增长9.4%；农村居民人均可支配收入7065元，增长12%。

一是脱贫攻坚步伐加快。制定“1+18”精准扶贫实施方案，组建帮扶工作队295个，完成贫困户6.65万户、28.6万人建档立卡和大数据平台建设。落实专项扶贫资金2.2亿元，实施整村推进项目53个。整合资金24.7亿元，集中用于贫困村基础设施和公共服务。发放涉农贷款252.7亿元，增长37.5%。全市行政村实现农民资金互助合作社机构全覆盖，贫困村农民资金互助合作社和引导资金双覆盖，政府投入引导资金9200万元。完成农村公路通畅工程757公里，解决129个贫困自然村三相动力用电问题，建设易地搬迁安置点9处。

二是工业经济运行平稳。推动传统产业改造升级，白银公司贵金属综合回收利用、刘化25万吨硝基复合肥、热电联产等项目投产，白银公司铜冶炼技术提升改造、锌冶炼资源综合利用、宏达铝业10万吨铝型材、生活垃圾焚烧发电等项目有序推进，华鹭铝业出城入园启动实施，煤炭、有色、电力、化工四大支柱行业拉动规模以上工业增长7个百分点。大力发展循环经济，实施循环经济项目142项，完成投资115亿元，我市被列为国家循环经济示范城市、餐厨废弃物资源利用和无害化处理试点城市，入围重金属污染防治中央财政专项资金支持城市。培育战略性新兴产业，高新技术企业总数达到26家，白银有色长通电线电缆公司和中科宇能入选全省战略性新兴产业总体攻坚战骨干企业，规模以上工业战略性新兴产业增加值完成27.8亿元，增长8.1%。

三是农业农村经济健康发展。稳定粮食产量，推广全膜双垄沟播139.4万亩、高效节水灌溉115.8万亩，农作物良种覆盖率达到95.1%，粮食产量达到85.4万吨，增长2.6%。发展特色农业，新改扩建日光温室1万亩，种植瓜菜96万亩，新增特色经济林15.7万亩，生猪、肉牛、羊饲养量分别达到174.8万头、16.2万头、486.6万只，肉蛋奶产量达到17.9万吨。加快农业产业化，新增农业龙头企业22家、专业合作社1017家，流转土地20万亩，发展规模经营73.4万亩，新认证无公害农产品产地17个、产品19个。实施农产品“出省入京”工程，实现销售收入26亿元。

四是第三产业增长加速。银东公铁联运工业物流园一期投运，中进仓储物流园一期主体封顶，鼎丰物流园保税仓和出口监管仓获兰州海关批准。建成市级特色产品展销馆暨电子商务运营中心、县区电子商务中心5个、乡镇服务站60个、村级服务点168个，会宁列入国家电子商务进农村综合示范县。黄河石林被列为全省重点建设的20个大景区之一，黄河假日城入选全国优选旅游项目名录，白银国家矿山公园建成，全市实现旅游综合收入42.5亿元。全省首家中外合资村镇银行中银富登入驻平川，甘肃菁茂公司成为我市首家、全省第3家“新三板”上市企业，甘肃股权交易白银运营中心揭牌。第三产业增加值增长8.7%，三产占生产总值比重提高4.7个百分点。

五是基础设施不断加强。抓住国家政策机遇，争取实施了一批基础设施建设项目。亚行二期、黄河干流白银段防洪治理、景泰黄崖坝土地整治、白银至中川机场一级公路、白银至景泰、靖远至会宁二级公路改建等项目开工，热电联产配套供热管网投运，白银城区防洪综合治理、中心街及片区棚户区改造等城市基础建设项目有序推进。成功进入全国首批地下综合管廊试点城市，并正式开工建设。

六是改革创新稳步推进。制定了户籍制度、城市公立医院、农业生产用水价格、居民生活用天然气阶梯价格等方面的改革措施。推进商事制度改革，完成“个转企”272户，发展市场主体1.7万户，非公经济增加值占生产总值比重提高到49.05%。组建PPP项目库，入库项目81个，总投资667亿元，16个项目开工实施，6个项目被国家发改委推介发布。参与设立兰白试验区技术创新驱动基金，新增省级重点实验室和工程技术研究中心各1家。科技企业孵化器被认定为首批国家小微企业创业创新示范基地，入驻企业116家，拥有专利149件,累计开发项目220个,“创客空间”投入运行，4家“众创空间”通过省级认定。启动建设碳纤维材料、精细化工等5个专业园区，银西生物医药园平整土地3800亩，6户企业洽谈签约。

七是民生事业持续改善。城镇新增就业6.54万人，输转劳务28.34万人，创劳务收入48.9亿元。全面实施机关事业单位养老保险改革,企业退休人员养老金、工伤人员伤残津贴、城市低保、失业保险标准均提高10%以上，城乡居民基本医疗保险（新农合）、基本养老保险政府补助标准分别提高到人均380元、85元。建成学前教育“939”项目幼儿园63个，消除中小学D级危房17.3万平方米，发放乡村教师生活补助1553万元，为584名符合条件的教师解决了职称遗留问题。景泰县、白银区、平川区通过国家义务教育均衡发展评估。市第一人民医院住院部完成主体建设，市儿童医院即将建成投用，省中医院制剂中心建成运行，我市列入全国城市公立医院综合改革试点。市食品检验检测中心建成运行。省、市确定的35件惠民实事全面完成。开展重点行业领域安全专项整治,安全生产形势稳定好转。落实党的民族宗教政策，促进了民族团结、宗教和顺。

与此同时，外事侨务、审计、统计、人防、双拥、档案、地方志、气象、红十字、老龄、妇女儿童等工作都取得了新成绩。

各位代表！“十二五”是我市经济总量快速提升、社会建设成效显著、人民群众得到实惠最多的时期。成绩来之不易，收获催人奋进，奋斗历程令人难忘。这些成绩的取得，得益于省委、省政府正确领导、亲切关怀，得益于市委统揽全局、科学决策，得益于市人大、市政协有效监督、全力支持，得益于全市人民和衷共济、团结奋斗，得益于社会各界关心帮助、通力协作。我代表市人民政府，向全市工人、农民、知识分子和各级干部，向驻银部队、武警官兵、公安干警和中央、省驻银企事业单位，向各民主党派、工商联、无党派人士、离退休老同志和社会各界人士，向在我市创业的境内外投资者，以及关心支持白银改革发展的朋友们，表示衷心的感谢和崇高的敬意！

各位代表！五年来的实践使我们深刻体会到：要实现白银经济社会平稳健康发展，必须坚决贯彻中央、省委的方针政策和决策部署，紧密结合白银实际，创造性地开展工作；必须用足用好国家扶持政策，把政策机遇转化为现实发展能力；必须坚持优化经济结构，不断提高发展质量和效益；必须大力发展循环经济，增强发展动能；必须秉承民生为本理念，让更多的群众共享改革发展成果。

我们也清醒地认识到，面对经济速度换挡、结构优化、方式转变、动力转换的态势，我们在主动适应和引领经济发展新常态、推动经济社会持续健康发展上还存在突出矛盾和问题。一是发展不足仍是最大市情，脱贫攻坚的“短板”问题比较突出；二是经济结构不优的矛盾还没有得到根本解决，转变发展方式的任务依然艰巨；三是财政收入增速放缓，刚性支出压力较大；四是城乡公共基础设施相对薄弱，园区聚集能力仍需提高；五是政府效能、服务水平还不高，部分干部干事创业、克难担当的精神还不够，发展环境仍需优化。矛盾蕴含机遇,问题就是动力。我们将坚持问题导向，进一步增强发展的紧迫感、责任感和使命感，科学谋划、勇于担当、扎实工作，努力把政府工作做得更好，不辜负全市人民的殷切期望和重托。

“十三五”经济社会发展主要目标和发展重点

“十三五”时期是全面建成小康社会的决胜阶段。为确保到2020年与全国一道全面建成小康社会，我们紧扣“四个全面”战略布局和“五位一体”总体布局，认真贯彻创新、协调、绿色、开放、共享的发展理念，根据《中共白银市委关于制定国民经济和社会发展第十三个五年规划的建议》，在广泛征求意见、集中各方智慧、充分研究论证的基础上，编制了《白银市国民经济和社会发展第十三个五年规划纲要(草案)》，分析了“十三五”时期的发展基础和环境，提出了经济社会发展的指导思想、发展定位、空间布局、主要目标、重大支撑工程及保障措施。

“十三五”主要预期目标是：生产总值年均增长8%，到2020年达到640亿元；一般公共预算收入年均增长8%；固定资产投资年均增长12%，五年累计投资3300亿元；社会消费品零售总额年均增长8%，达到250亿元；居民消费价格指数控制在103以内；城镇居民人均可支配收入增长7.5%，农村居民人均可支配收入增长9.5%，分别达到33648元和11122元。

以上目标，主动适应和引领经济发展新常态，全面贯彻供给侧结构性改革新要求，综合考虑了小康目标和可行可为的统一，当前工作和长远发展的统一，质量、效益、结构、速度的统一，经济发展和改善民生的统一。从发展要求看，实现这一目标，可确保2020年国内生产总值比2010年翻一番以上，全面建成小康；从发展基础看，“十二五”我市在产业结构调整、转型升级、“3+4”产业集群、园区建设等方面打下了坚实基础，投资效应将在“十三五”得到释放；从发展机遇看，国家“一带一路”、新一轮西部大开发、脱贫攻坚战略的实施，以及资源型城市转型、大兰州经济区、兰白科技创新改革试验区建设等，将对我市发展和经济增长起到重要的拉动作用。目标的制定积极务实，既是必要的，也是可行的。

按照《纲要》（草案）要求，“十三五”时期，我们将以创新驱动为主线，以改革开放为动力，以资源型城市转型为主要路径，以改善民生为落脚点，实施“八大战略工程”，建设“一市五区七基地”，全力打造产业特色鲜明、城乡功能完善、生态环境优美、社会和谐稳定的繁荣文明新白银。发展重点主要集中在以下四个方面:

一、突出脱贫攻坚，与全国一道全面建成小康社会

全面建成小康社会是“十三五”时期最大的政治任务。我们必须坚持以精准扶贫、精准脱贫为主要抓手，聚焦重点区域，破解发展的公共性、基础性问题，确保实现整体脱贫。

改善农村基础条件。以三个六盘山片区特困县为主，加快推进引洮会宁供水、新一轮大中型灌区续建配套与节水改造等骨干水利工程，实施甘肃中部生态移民扶贫开发供水、兴电灌区扩容改造、景电三期景泰片区电力提灌等工程，开发整治土地10万亩，建设全省生态移民示范区。完善贫困村“水、电、路、房”等基础设施，加快整村推进，实现自然村动力电全覆盖，全面完成建档立卡贫困农户危房改造，构建从“源头”到“龙头”的农村饮水安全体系。

发展农村富民产业。构建“一带两区”特色农业区，以沿黄灌区为主，发展设施养殖、优质瓜菜、现代制种等产业，打造高效农业示范带；以高扬程灌区为主，发展节水种植、草食畜牧、特色林果等产业，建设立体复合型农业经济区；以南部旱作区为主,做大做强马铃薯、小杂粮、玉米、饲草等产业，大力发展绿色农业，建设旱作特色农业经济区。实施“四个100万亩”和“两个高效”工程。依靠国家农业科技示范园区辐射带动，创建现代农业示范园（区）20个、标准化规模种植基地30个、养殖场（小区）300个，新增农业产业化龙头企业50家，农产品加工转化率提高到60%。

推进公共服务向农村延伸。完善县、乡、村三级金融服务网络，扩大精准扶贫小额信贷、易地扶贫搬迁贴息贷款、惠农“三项贷款”、农民资金互助合作社借款规模，有效解决贫困户发展资金难题。加强扶贫政策与农村低保制度有效衔接，提高农村养老保险、新农合政府补助标准和五保对象供养水平，加快养老福利机构、乡镇敬老院和社区(村)老年人日间照料中心建设，健全农村留守儿童和妇女、老人关爱服务体系。

二、突出创新驱动，加快产业结构调整和转型升级

创新是“十三五”发展的第一动力。我们必须坚持把发展基点放在创新上，以兰白科技创新改革试验区建设为统领，深入实施创新驱动战略，把创新贯穿于转型发展的各领域、全过程，构建产业新格局。

大力推进兰白科技创新改革试验区建设。以白银工业集中区“一区六园”为载体，加强与上海张江等园区合作，聚集创新要素，塑造发展优势。构建创新体系，建成国家级重点实验室和企业技术中心各1家，省级工程技术研究中心10家，培育高新技术企业40家，科技型企业200家；成立白银新材料研究院，新组建产业技术创新联盟2个。加快科技成果转化，建成科技企业孵化器8家,国家级孵化器2家。推动大众创业、万众创新，建成省级创业就业孵化示范基地（园区）5个，积极争创国家小微企业创新创业示范城市；投放创业担保贷款15亿元以上，筹措担保基金9000万元，用于扶持创业创新。实施商标品牌培育战略，创建国家知识产权示范城市，专利申请量年均增长20%以上，万人发明专利拥有量达到4.5件。全社会研发投入占生产总值的比重达到2.5%，科技对经济贡献率达到55%。

培育壮大战略性新兴产业。立足产业基础和发展潜力，主攻新材料及制品、新能源及配套、新型煤化工、先进装备制造等产业，战略性新兴产业增加值占生产总值比重提高到20%。以有色金属新材料、稀土功能材料、碳纤维材料、凹凸棒石材料、硅材料为重点，建设西部有色金属新材料基地。以煤气化联产技术为核心，发展煤制合成气、煤制芳烃和烯烃，实现煤炭资源就地转化增值和循环利用。以兆瓦级叶片、非晶合金变压器、内馈节能电动机、新型电线电缆和超导电缆、矿用设备为主，发展专用设备、电气机械及器材、大型采矿设备等装备制造业，提高规模和质量。加快生物医药、节能环保、电子信息等技术密集型产业发展，推动产业由资源主导型向科技主导型转变。

改造提升传统产业。坚持走新型工业化道路，推进传统产业中高端化，延伸产业链条，提高产品附加值，工业增加值年均增长8%。有色金属产业，新增铜冶炼产能30万吨，铜铅锌冶炼产能达到130万吨以上；化工产业，建设银东循环化工园，壮大以TDI为核心的光气化产业链，打造全国最大的含能材料生产研发及聚氨酯产业基地；能源产业，新增火电装机350万千瓦、水电装机68万千瓦，总装机达到1050万千瓦，形成煤炭、火电、水电、风电、光伏发电并举的综合型能源产业体系，建设西部综合能源开发转化消纳基地；陶瓷建材产业，重点发展中高端陶瓷制品、新型墙体材料，建设西部陶瓷建材产业基地。推进国家循环经济示范城市建设，构建覆盖全社会的资源循环利用体系。

加快发展现代服务业。同步发展生产性服务业和生活性服务业，实现第三产业增加值年均增长9%，占生产总值比重达到44.5%。坚持“服务带动、物流先行”原则，构建“一个枢纽、两大基地、五个聚集区”现代物流总体格局。积极融入“兰州-白银-中卫-银川”黄河风情旅游经济带、“兰州-白银-庆阳-延安”红色旅游经济带和“白银-河西五市”丝绸之路旅游经济带。推动多种所有制金融机构共同发展、互相补充，新增金融机构3家以上，实现村镇银行县区全覆盖。培育电子商务交易平台，建设电子商务创业园区,开展电子商务进农村综合示范工作。鼓励社会资本兴办老年公寓、养老院、护理院等养老设施，构建以居家养老为基础、社区服务为依托、机构养老为补充的养老服务体系。

以改革开放释放创新活力。深化国有企业改革，推进市城投公司、水务集团、交通投资公司等投融资类企业融资手段创新。创新政府投资方式，探索设立产业投资基金和创业投资基金，推广PPP项目融资机制。提高对外开放水平，立足“丝绸之路经济带”重要节点城市区位优势，鼓励企业与东中部产业转移项目合作，建设“丝绸之路经济带”国际产能合作区。

三、突出夯实基础，增强发展承载功能

基础设施是“十三五”发展的重要保障。我们必须按照统筹规划、合理布局、适度超前的原则，加快交通、水利、信息、管网等建设，着力补齐基础设施“短板”。

构建区域交通体系。提升公路等级，改造国省道，畅通县乡道，新改建公路4762公里，基本形成连接通畅、高效便捷的市域公路体系。配合实施包兰铁路中卫至兰州客运专线、干武铁路增建二线工程，推进环海中、白银至平凉、环兰州城际会宁至平川段等铁路连通工程，构建“三横三纵”铁路交通网络，打通白银东西、南北铁路连接通道。建设白银支线机场，扩大交通辐射半径。

提高城市建设水平。完善城区道路网，建设市区道路17条。抓好地下综合管廊试点项目，逐步实施平川、会宁、靖远、景泰城区地下管廊工程。完善供水、供热、供气管网体系。创建“国家级园林城市”，人均公共绿地面积达到10平方米，绿化覆盖率达到40%以上。加快城镇污水处理、垃圾无害化处理设施建设，污水处理率达到90%，垃圾无害化处理率达到95%。推进老旧城区、“城中村”、城乡结合部棚户区和危房改造，城镇常住人口保障性住房覆盖率达到20%左右。加强停车场、公共厕所等公共设施建设，方便市民生活。加快省、市、县各级新型城镇化示范镇及部分重点乡镇基础设施建设，培育成为特色小城镇。全市城镇化率达到53%。

提升信息基础设施。积极创建“宽带中国”示范城市，优化升级宽带网络，推进光纤入户。加快新一代移动通信网络建设，推动“三网融合”，统筹推进“智慧城市”、“信息消费”、“信息惠民”工程建设，打造云计算数据中心。全市固定互联网宽带接入用户达到35万户，通信运营企业固定宽带接入能力超过50万户，互联网城域出口带宽达到500G以上。

构筑生态安全屏障。围绕国家生态安全屏障综合试验区建设，以瞭高山、寿鹿山、哈思山、屈吴山、铁木山为主建设生态保护区，以风沙危害区、水土流失区、土地盐碱区、工业破坏地恢复区为主建设生态治理区，以人口聚集区域为主建设生态优化区。新增造林封育面积160万亩，森林覆盖率达到15.1%。推进美丽乡村建设，实施千村美丽万村整洁、乡村环境绿化美化工程。加强重点流域综合治理，新增水土流失治理面积632平方公里，新修梯田44万亩。推进以大气、水、土壤为重点的环境治理，二氧化硫、氮氧化物、化学需氧量、氨氮排放量、可吸入颗粒物等主要污染物指标进一步下降。

四、突出民生改善，促进共享发展

改善民生是“十三五”发展的落脚点。我们必须坚持共享发展理念，进一步加大民生投入，着力增进人民福祉。

多渠道增加就业。全面落实就业扶持政策，帮助困难群体就业，引导高校毕业生到基层特别是到非公经济组织就业。加强新生劳动者就业技能培训，提高劳动者就业竞争力。新增就业20万人以上，城镇失业人员再就业3万人，城镇登记失业率控制在4%以内。

推进教育均衡协调发展。大力发展学前教育，60%以上的乡镇中心幼儿园实现标准化办园，有学前教育需求的行政村实现幼儿园全覆盖。推进义务教育均衡发展，确保适龄儿童免费就近入学。鼓励普通高中与职业学校资源共享。加快发展现代职业教育，整合优化职业教育资源。努力推进高等教育发展，争取建设1-2所综合性大学分校。学前教育毛入园率达到85%以上，义务教育巩固率达到99.8%以上，高等教育毛入学率达到60%以上，主要劳动年龄人口平均受教育年限达到11.5年，新增劳动力平均受教育年限达到14年。

优化基本医疗卫生服务。加快健康白银建设，统筹推进医疗保障、医疗服务、公共卫生、药品供应、监管体制综合改革，建立覆盖城乡居民的基本医疗卫生制度。坚持公立医院公益性质，全面推进市县公立医院综合改革。鼓励社会办医，大力发展健康服务业，优化医疗卫生资源布局，健全上下联动、衔接互补的医疗服务体系，促进医疗资源向基层、农村流动。

健全公共文化体系。实施农村文化惠民工程，改造提升乡镇文化站9个，所有行政村建成“乡村舞台”。强化公共文化产品供给能力，实现市、县、乡、村四级公共文化服务网络全覆盖，更好地保障群众基本文化权益。

提高社会保障水平。完善养老保险、医疗保险、住房保障、社会救助和福利体系。实施全民参保计划，基本实现法定人员全覆盖。落实渐进式延迟退休年龄政策。加强保障房建设，解决住房困难群众和进城落户农民住房问题。健全社会救助和保障标准与物价上涨联动机制。巩固低保“应保尽保”成果。稳步推进收入分配制度改革，加快提高城乡居民收入水平。

2016年政府重点工作

2016年，是实施“十三五”规划的开局之年，也是推进结构性改革的攻坚之年。全市经济社会发展主要预期目标是：生产总值增长7.5%，固定资产投资增长11%，社会消费品零售总额增长8%，一般公共预算收入增长6%，城镇居民人均可支配收入增长10%，农村居民人均可支配收入增长10.5%，城镇登记失业率控制在4%以内。重点抓好以下工作：

一、抓精准施策，促进脱贫攻坚

加大精准扶贫力度。完善“1+18”精准扶贫方案，研究制定脱贫攻坚专项规划。整合各类涉农资金，统筹支持脱贫攻坚，落实片区县按当年一般公共预算收入增量20%、插花县15%、市级10%以上增列专项扶贫预算的政策，建立市级财政专项扶贫资金竞争性分配机制。坚持对口帮扶常态化，开辟精准扶贫建设项目绿色通道,建立精准扶贫项目优先办理、“容缺补办”和限时办结等机制。完善精准扶贫大数据平台，加强动态管理，落实退出机制。规范农民资金互助合作社运行管理，力争贫困村政府引导资金增加到50万元。实施易地扶贫搬迁工程，完成搬迁465户、2194人。确保7.15万贫困人口脱贫，平川区实现整体脱贫。

继续调整农业结构。推广旱作农业技术142万亩、测土配方施肥444万亩、高效节水115万亩。创建高产万亩示范片20个，粮食总产量稳定在75万吨以上。做强做优“高原夏菜”，蔬菜种植稳定在30万亩以上，总产量保持在130万吨以上。新增特色经济林12万亩。新改扩建标准化规模养殖场（小区）70个，肉蛋奶总产量稳定在18万吨以上。

加力推进农业产业化。聚焦国家农业科技园区建设，完善园区建设规划，发展设施蔬菜、精深加工、物流信息产业，打造黄河上游旱作补灌区绿色农产品生产、加工和流通基地。提高农业生产经营组织化程度，新增农业产业化龙头企业10户，农产品加工转化率达到53%，市级以上农民专业合作示范社达到450家。发展品牌农业，创建国家级、省级知名品牌产品3个，认证无公害产地10个。继续推进农产品“出省入京”工程。建设农产品质量安全追溯平台，创建靖远县国家级和平川区省级农产品质量安全县（区）。

二、抓挖潜提升，强化工业主导

着力调整产业结构。把握供给侧结构性改革新要求，以白银公司、银光公司、靖煤集团等骨干企业为重点，积极推广应用新技术、新工艺、新装备，支持企业化解过剩产能。改造提升有色金属、化工、能源等传统产业，重点抓好华鹭铝业出城入园、重庆昌元化工重铬酸钠配套清洁生产、中瑞铝业铝合金、靖煤集团刘化合成氨及尿素、甘肃华盛新工艺芳烃等项目建设，实现规模以上工业增加值增长5.5%。争取省战略性新兴产业创业投资基金，支持甘肃稀土新材料、白银有色长通电线电缆、中科宇能、郝氏碳纤维等高新技术企业发展壮大，力争战略性新兴产业增加值增长15%以上。

着力帮助企业降低成本。高度重视困难企业扭亏脱困工作，帮助企业降低人工、电力、物流、社会保险、融资、财务等成本，为实体经济让利。对连续亏损三年且不符合结构调整方向的“僵尸企业”，采取资产重组、产权转让等方式予以“出清”。进一步争取扩大大用户直购电交易范围和规模，加快兰白地区微电网建设前期工作。加强企业与金融机构对接，延长中小企业贷款期限，促进下滑企业止滑稳增、停产企业尽快复活，形成良好的市场预期。落实各项优惠政策，支持非公企业发展。

着力推进“两化”融合。按照企业“两化”融合管理体系国家标准，开展试点工作,争取1-2户大中型企业实现“两化”融合等级跃升。建设中小企业信息化服务平台，制定推进“互联网+”、“中国制造2025”工作政策措施，以信息化促进工业化。

着力发展循环经济。推进高新区循环化改造，加快聚能达树脂、扎布耶锂业二期等15个项目建设。推进资源综合利用“双百工程”示范基地建设，培育循环经济骨干企业35户,建成餐厨垃圾资源化利用和无害化处理项目。推广农作物秸秆还田、生物饲料、饲料加工等综合利用技术，秸秆综合利用率达到81%，废旧地膜回收利用率达到80%以上，尾菜处理利用率达到35%。

三、抓项目支撑，扩大有效投资

抓好重点项目建设。继续实行领导负责制、部门跟踪服务制和一线工作法，实施市列重点建设项目64个，年度投资达到150亿元。重点实施中核钛白20万吨钛白粉资源综合利用、熙瑞生物菊粉加工扩建等项目，确保白银公司铜冶炼技术提升改造、锌冶炼资源综合利用、甘肃长盛废旧汽车回收拆解、生活垃圾焚烧发电等项目建成投产。

精心谋划引进项目。根据国家产业政策、发展趋势，深入谋划一批重大基础设施、战略性新兴产业和现代服务业项目，扩大项目库容。重点推进煤化工、石油化工、黑山峡梯级开发、中电国际白银实验性太阳能示范等重大项目前期工作。围绕“3+4”产业基础、围绕“三个500强”企业，引进一批重点企业和上市公司来银投资，引导白银籍在外创业、务工有成人员回乡投资兴业，落实签约招商项目“帮办制”，建立履约推进责任制，提高招商项目履约率。

拓宽项目融资渠道。发挥政府投资“四两拨千斤”作用，撬动社会资本参与项目建设，推广PPP模式，加快实施16个已开工的项目，尽快开工20个已开展前期工作的项目，争取更多项目入库落地。扩大直接融资规模，加大企业债券融资力度，鼓励引导符合条件的企业争取专项债券。完善金融服务网络，争取浦发银行、锦州银行在银设立分支机构，兰州银行、中银富登村镇银行分别在景泰、靖远设立分支机构。支持创新型中小微企业在“新三板”上市。加强融资担保体系建设，规范发展小额贷款公司和融资性担保公司。充分发挥市城投公司、水务集团、交通投资公司、黄河石林开发公司等政府融资平台作用，盘活优质国有资产，增强融资能力。

四、抓兰白试验区建设，推进自主创新

突出企业主体地位。新增科技型企业15家、高新技术企业3家，争取一批科技型企业进入“小巨人”企业。加快重点企业创新载体建设，建成省市级工程技术中心5家以上。建设国家知识产权试点城市。提升专利申请质量，提高发明专利授权量，万人发明专利拥有量达到1.7件。

提升园区创新能力。加快高新区产业孵化创新基地建设，实施巨亨创新园（加速器），新建出孵企业产业化项目5个，配套建设专家人才公寓，打造集文化、研发、公共服务等为一体的高端孵化社区平台。争取刘川工业园升级为省级园区。以“3+4”产业集群为重点，合理布局白银工业集中区“一区六园”产业，规划建设白银氟化工产业园，推进生物医药、碳纤维材料、精细化工、新能源产业装备制造、稀土新材料五个专业园区建设，促进产业集约化发展。力争“一区六园”工业增加值占全部工业增加值比重达到70%以上。

拓展科技合作交流。争取高校科技园在银建立分园、兰州大学建设化学技术研究院，推进县区孵化器建设。争取并利用好兰白试验区技术创新驱动基金、省科投公司科技企业孵化器专项基金、科技投资基金和科技创新创业投资引导基金；推进与张江高新区、上海产业技术研究院合作，建设生物医药产业化基地、3D打印和水处理应用中心。加强创新人才建设，实施“铜城学者”和产业创新团队计划，支持企业对有特殊贡献人才实行股权奖励、技术入股等激励措施。

五、抓基础提升，着力破解发展瓶颈

加快构建交通路网。推进包兰线中卫至兰州客专白银段、景泰（营盘水）至中川高速公路、国道341线白银至中川机场一级公路、国道338线景泰段建设，加快国道247线靖远至会宁段、国道312线会宁城区过境段、省道217线白银至景泰段公路改造。做好景泰至靖远至会宁高速公路、环海中铁路、白银支线机场、通航机场等项目前期工作。新建农村公路1100公里,实现全市建制村通沥青(水泥)路。

加强水利基础设施建设。推进黄河干流白银段防洪治理、引洮一期会宁北部供水、大型泵站更新改造、祖厉河城区综合治理二期、靖会干渠甘沟延长段等水利项目，配合实施引洮二期会宁片区工程，发展有效灌溉面积2万亩，兴修梯田12万亩，水土保持综合治理140平方公里，解决8.18万人饮水不安全问题。

改善城市基础条件。完成城市总规编制,修订控制性详细规划、村镇规划,编制片区控规、城市城镇风貌、城市夜景照明等专项规划。加强城市路网建设,新建上海路北段、凤凰街、北环路西段等5条道路9.4公里；修缮改造城区道路,提高道路通行能力。健全垃圾污水处理、供热供电、供水排水等配套设施，加快城市地下综合管廊建设,开工建设南环路等6条主管廊20.7公里。实施国道109线过境段绿化、长通公园景观改造等园林绿化工程。积极推进“智慧城市”建设,加大无线宽带网络信息基础设施建设力度,推动物联网、大数据等新一代信息技术应用。加快城市管理平台和市民卡建设。完成信息基础设施投资5.5亿元。

六、抓规模扩张，发展现代服务业

提速发展商贸物流业。加快银西商贸物流园、银东工业物流园建设，抓好大西北仓储式物流园区、汽车商贸服务城建设，辐射带动“五大物流集聚区”一体发展。开行“白银号”中欧国际货运班列，建成银西综合保税物流园、居然之家白银阳明广场。加快大型商品交易市场建设，发展社区综合性便民服务。扎实推进“粮安工程”，保障粮食市场供应和价格基本稳定。

融合发展文化旅游业。启动国家公共文化服务体系示范区和会师旧址、黄河石林5A级景区创建工作，实施丝绸之路黄河文化遗产博览城建设项目。建设市文化艺术中心,加快红军长征胜利景园、永泰古城、大敦煌影视城、水川湿地公园、靖远腾飞体育文化产业园、会宁汉唐二十四节气文化休闲商业街建设。游客人数、旅游综合收入各增长20%以上，文化产业增加值增长10%。

壮大发展电子商务。以国家和省级电子商务进农村综合示范县建设为契机，对接阿里、京东、苏宁易购等知名电商企业，加快邦农快件物流园、靖远电商产业园、会宁“九馆一中心”、景泰电商孵化园等项目建设，电子商务交易额增长30%以上。

七、抓民生工程，改善城乡居民生活条件

大力改善人居环境。实施燃煤电厂超低排放改造、燃煤锅炉治理提标改造、东大沟重金属污染综合治理以及历史遗留含铬土壤污染治理等工程，积极推进园区污水处理项目建设，加强饮用水源地保护，强化城市环境综合整治，提升环境质量。打造省市县梯级美丽乡村示范村25个。完成退耕地造林5.3万亩、农田林网2万亩、大环境绿化1万亩、天然林缘区人工造林1万亩、村庄美化4.5万亩，森林覆盖率达到14.4%。继续开展“五城联创、六城同建”活动，提高城市管理水平和市民文明素质。

统筹发展社会事业。实施“全面改薄”工程，建设农村小学附设幼儿园338所；向贫困村中小学、幼儿园教师发放生活补助，对学前教育幼儿免保教费，免除建档立卡贫困家庭普通高中学生学杂费和书本费；组建白银职业教育集团，推进国家开放大学白银分院建设，争取建设甘肃旅游学院、兰州城市学院白银分院。实施一对夫妇可生育两个孩子政策。提升市县公立医院服务能力,建成市第一人民医院住院部,实施市全科医生培训基地、市妇幼保健院、会宁县人民医院等项目。完成市民中心主体建设。

提高社会保障水平。设立创业带动就业扶持资金，加大对就业创业工作支持力度，新增城镇就业4.1万人。全面实施全民参保登记计划，继续扩大参保覆盖面。实施“同舟计划”，推进建筑业等高风险行业参加工伤保险。完善城乡居民基本养老保险制度，续保率达到90%以上，养老金发放率达到100%。推进城镇居民医保和新农合两项制度整合工作，进一步提高筹资水平。全面实施城乡居民大病保险，继续推进异地就医结算。有效化解房地产库存，做好存量商品房回购工作，加大棚户区改造货币化补偿安置力度，努力将货币化补偿安置率提高到50%以上。改造棚户区5333户（套），建设公租房1908套。推进中心街和八号楼旧城改造，完成老旧住宅整治7344户。

继续兴办惠民实事。坚持兴办惠民实事制度化，集中财力解决一批事关群众切身利益的突出问题。市政府在广泛征求意见的基础上，根据财力实际，确定兴办“10件惠民实事”：（1）建设县乡农贸市场10个；（2）建成“乡村舞台”146个；（3）建设“一村一场”农民体育健身工程100个；（4）建成77所标准化村卫生室，实现全市标准化村卫生室全覆盖；（5）新型农村合作医疗筹资标准提高到530元；（6）通过“淘宝‘云客服’残疾人批量就业项目”培训残疾人500名，实现上岗就业200名；（7）完成农村危房改造1.15万户；（8）白银城区65岁以上老年人免费乘坐公交车；（9）城市低保标准提高10%，提高农村低保一、二类标准，实现与扶贫脱贫线“两线合一”；（10）新建环境整洁村138个。

加强政府自身建设

新形势、新任务对政府工作提出了新的更高的要求，我们必须不断适应新的形势，加强政府自身改革，努力建设为民务实、高效廉洁、群众满意的政府。

严格依法行政。健全依法决策机制，“三重一大”事项坚持公众参与、专家论证、风险评估、合法性审查、集体讨论决定，推行重大决策终身责任追究、责任倒查、跟踪反馈和纠错问责制。完善法律专家咨询委员会、法律顾问、行政复议等制度。抓好“七五”普法工作，加强公共法律服务体系建设，扩大公共法律服务覆盖面。深化行政执法体制改革，推动执法重心和执法力量下移。自觉接受人大及其常委会监督，主动接受政协民主监督，重视舆论、社会监督，保障公民各项权益。

加快转变职能。继续取消调整下放一批行政审批项目,把简政放权、放管结合、优化服务推向纵深。完善“四单一网”,创新监管方式，建成全市在线审批监管平台,实现市县政务服务网互联互通，推进重点领域信息公开。完善并联审批，推进公共资源电子化招投标，加快中介服务中心建设，清理规范中介组织。深化事业单位分类改革，推进生产经营类事业单位转企改制。

创新社会治理。深化社会组织管理改革，加快行业协会、商会与行政机关脱钩。坚持网格化管理、社会化服务方向，健全基层综合服务管理平台，推动政府治理和社会自我调节互动。健全公共安全防控体系，推进“平安白银”建设。深入开展安全生产专项整治行动，健全安全生产社会共治体系，做好园区规划和危化项目的安全评价工作。强化信访问题源头治理，妥善化解信访热点难点问题。加强食品、药品、农产品等市场监管，规范市场秩序，严厉打击假冒伪劣、坑蒙拐骗等违法犯罪行为。加快推进信用白银建设，创建国家信用示范城市。加强防震减灾工作，推进国家防震减灾示范城市建设。

切实改进作风。弘扬学习调研之风，推动政府人员加快知识更新、优化知识结构。加强政务督查、效能监察和行政问责，大力整治“为官不为”、“懒政怠政”等现象。自觉践行“三严三实”，切实履行“一岗双责”。加强对重要领域、关键岗位的行政监察和审计监督，严控“三公”经费，确保公共资金安全。

各位代表！改革开启新征程，时代赋予新使命。让我们紧密团结在以习近平同志为总书记的党中央周围，在省委、市委的坚强领导下，以更加坚定的信念、更加务实的作风、更加饱满的热情，迎难而上，埋头苦干，为谱写白银人民美好生活新篇章而努力奋斗！
